# Exploring the different models of the four-day workweek as social innovations in Germany’s healthcare system

**DOI:** 10.1186/s12913-026-15099-5

**Published:** 2026-07-14

**Authors:** Katharina Hast

**Affiliations:** Sociology of Work and Organization, Department of Social Sciences, TU Dortmund University, Emil-Figge-Str. 50, 44227 Dortmund, Germany

**Keywords:** Social innovation, Organizational change, German healthcare system, Four-day workweek, Healthcare workforce, Nursing shortage

## Abstract

**Background:**

The German healthcare system faces a persistent shortage of skilled professionals, particularly in nursing, driven by demographic change, high workloads, and challenging working conditions. In response, new working-time models such as the four-day workweek have emerged as potential solutions to improve job attractiveness, employee well-being, and staff retention. This study examines different models of the four-day workweek as social innovations within the healthcare system, focusing on their development, implementation, and implications across institutional levels.

**Methods:**

A qualitative research design was employed, with 27 semi-structured interviews conducted between August 2024 and February 2025. Participants included nurses, physicians, hospital managers, political stakeholders, and representatives from unions and professional organizations. Data were analyzed using qualitative content analysis following Mayring, supported by MAXQDA software. The analysis was guided by a theoretical framework on the institutionalization of social innovations, examining processes across micro-, meso-, and macro-levels. An iterative approach was used to link empirical findings with theory.

**Results:**

Three main working-time models were identified: part-time work, the compressed four-day workweek (constant weekly hours distributed over fewer days), and the reduced four-day workweek (fewer hours with unchanged pay). Part-time work is widely used as an individual coping strategy but entails financial risks. The compressed model is more easily implemented within existing regulatory and financial structures and is perceived as attractive, yet it does not significantly reduce workload and may intensify daily strain. The reduced model shows greater potential for improving well-being and retention but faces substantial challenges related to staffing, financing, and regulatory constraints. Overall, findings reveal tensions between normative expectations of high-quality care and limited resources, leading to paradoxical outcomes across system levels.

**Conclusions:**

The compressed four-day workweek represents an adaptive but limited innovation, while the reduced model holds transformative potential but requires systemic change. Effective implementation of new working-time models in healthcare depends on coordinated action across institutional levels, including supportive financial, regulatory, and organizational frameworks.

**Supplementary Information:**

The online version contains supplementary material available at 10.1186/s12913-026-15099-5.

## Background

The German healthcare system is confronted with a critical shortage of healthcare professionals, a challenge shared by other Western health systems. This phenomenon is, at least in part, attributable to the aging population. An aging population signifies a rise in the number of individuals requiring medical assistance, while concurrently diminishing the labor force [1–3]. Simultaneously, the scarcity of skilled labor and the consequent accumulation of workloads have engendered a deleterious downward spiral. For instance, a significant proportion of nurses work part-time [4]. Consequently, the remaining healthcare professionals face an increased workload. Employees experience chronic stress, burnout, and low job satisfaction [5, 6]. Frequent night shifts and irregular working hours have been demonstrated to have a negative impact on employees’ work-life balance. Furthermore, the existence of gaps in training, limited career opportunities, and a disconnect between policy decisions and the reality of the workplace contributes to further dissatisfaction [7–12].

In Germany, healthcare facilities are implementing new work schedules with the objective of enhancing the appeal of these professions for their employees. Some hospitals [13] have introduced a compressed four-day workweek (C4DW) for their nursing staff. This means that the weekly working hours remain the same, but are now spread over four days. Salaries remain unchanged. Furthermore, there are hospitals [14] and nursing homes [15] that offer or would like to offer a reduced four-day workweek (R4DW) for all employees. In this context, an R4DW means that the weekly work hours are reduced, usually by 20%. However, he salary remains unchanged despite the reduced hours, which effectively corresponds to higher hourly pay. The introduction of various forms of the four-day workweek (4DW) in the healthcare sector in recent years may be due to the significant attention the R4DW has received in scientific studies and public discourse [16–29].

A previous analysis examined the extent to which stakeholders in the healthcare sector view an R4DW as sustainable and which values and norms play a particular role in this process. A distinction was made between economic, social, and “social performance” sustainability [30].

In this article, an examination of the C4DW and the R4DW for nursing staff is conducted, along with an analysis of their development in recent years. The primary focus of this study is on the adaptation to the system, the potential benefits for organizations and employees, and the challenges involved. To provide a comprehensive overview, it is necessary to consider a third model that is frequently observed in practice: part-time employment. A significant proportion of the nursing staff have been observed to reduce their working hours in order to cope with the high workload, which is why part-time work is a central component of their work reality. The analysis deliberately focuses on nursing staff, as they are at the center of the debate surrounding the compressed 4DW.

To analyze the various forms of the 4DW, the present study draws on a model by Cajaiba-Santana [31] that illustrates the institutionalization process of social innovations. Social innovations are defined as new, institutionalized, and socially accepted practices. Their objective is to address needs and problems that have yet to be adequately resolved. Innovation can be categorized into various types, including technical, social, sustainable, and organizational innovations. Social innovations are defined as novel practices or behaviors that effectively address challenges in a manner that was previously unattainable. In this context, the term “new” is relative to the respective field of action [32].

Organizational innovations constitute a subcategory of social innovations, predominantly operating at the meso level. Such practices represent novel modes of corporate conduct, exhibiting a favorable influence on both the corporation’s effectiveness and the well-being of its workforce [33]. The perception of social innovations and their subsequent effects can be characterized by a sense of ambivalence, which can evolve over time. Cajaiba-Santana integrates new institutionalism and structuration theory to explain social innovations and their institutionalization process. To this end, he draws upon both structuration theory and new institutionalism. In an iterative cycle, an agent develops an idea that institutions either accept or reject. The institutions then legitimize the agent’s social practices. Social practices take place within groups, between groups, and across society as a whole. These social practices, in turn, are reflected upon by the agent [31].

The 4DW is considered a social innovation only through processes of translation, adaptation, and institutionalization [31]. Therefore, the objective of this paper is to examine the models of the 4DW as a potential social innovation and to address the following questions:

### RQ1

*How is the 4DW constructed, translated, and adapted as a social innovation across micro-, meso-, and macro-levels in the healthcare system?*

### RQ2

*What paradoxes and limitations arise as the 4DW is translated into organizational practice under conditions of high regulation and resource scarcity?*

## Methods

### Study design

A qualitative research design was developed to analyze how the 4DW models are integrated in the healthcare system and what developments might arise. Germany’s healthcare system poses significant challenges to innovation due to its stringent regulations, collective financing structure, and persistent workforce shortages [34].

A preliminary step in the research process involved conducting a literature review of scientific and non-scientific articles. This was done to develop a more comprehensive understanding of the discourse surrounding the 4DW in the healthcare sector. Subsequently, two interview guides were developed for semi-structured interviews. One was aimed at all stakeholders in the healthcare sector in general, and one was specifically designed for nursing staff. Depending on the group of actors, the questions focused on the macro, meso, and/or micro levels.

In order to comprehend the 4DW within its institutional context, it was imperative to survey a diverse array of healthcare stakeholders. This included stakeholders who work directly under various work schedules (nurses) and those who work alongside this professional group (e.g., physicians). The objective of the study was to understand management decisions, which is why nursing directors, nursing coordinators, nursing service managers, and works council representatives were interviewed. The political level was addressed by stakeholders representing trade union, political, and professional interests. These include politicians active in the nursing sector, union representatives of nursing staff and physicians, representatives of the Baden-Württemberg Nursing Chamber, representatives of health insurance companies, and works council members. Some participants who are now active at the macro level have also worked in hospitals and thus gained experience at the micro level. The table provides a simplified overview of the different levels. At the same time, some people are active at both the micro and meso levels simultaneously, for example, as a physician and a member of the works council. The objective of the interviews was not to provide a comprehensive and representative overview of each group; rather, it was to understand the processes within the healthcare system and to grasp how these processes are interconnected at the micro, meso, and macro levels. In principle, efforts were made to conduct the interviews in person to ensure a comfortable conversational atmosphere. In instances where in-person interaction was not feasible, the interviews were conducted via Zoom. The interviews were conducted between August 2024 and February 2025. The subsequent table (Table 1) presents a comprehensive overview of the interviewees. A total of 27 interviews were conducted, with each interview lasting between 49 min and one hour 50 min.

**Table 1:** Interviewee details

Level	Interviewee	Gender
macro level	State Parliament Member with a Focus on Health and Social Policy 01	male
	State Parliament Member with a Focus on Health and Social Policy 02	male
	State Parliament Member with a Focus on Health and Social Policy 03	male
	State Parliament Member with a Focus on Health and Social Policy 04	female
	State Parliament Member with a Focus on Health and Social Policy 05	female
	Federal Parliament Member with a Focus on Health and Social Policy	male
	Representative of Nursing Chamber	female
	Trade Union Secretary 01	male
	Trade Union Secretary 02	male
	Trade Union Secretary with a Focus on Youth Issues	male
	Policy Officer for Nursing Policy, Health Insurance Provider	male
	Representative of a Health Insurance Provider	male
meso level	Director of Nursing 01	male
	Director of Nursing 02	female
	Director of Nursing 03	male
	Director of Nursing 04; Head of Nursing Services	male; female
	Nursing Expert from a Non-profit Provider Organization	male
	Chairperson of a Regional District Association of a Non-profit Provider	male
	Chief Physician	male
meso and micro level	Staff Council Representative and Physiotherapist	male
	Staff Council Representative and Physician 01	male
	Staff Council Representative and Physician 02	male
micro level	Nurse and Youth/Apprentice Representative in the Staff Council	female
	Nurse 02	female
	Nurse 03	female
	Nurse 04	male
	Nurse 05	female

Institutionalism and structuration theory serve as the theoretical basis for the interview guides. The research heuristics were based on the integration of these theories as proposed by Wilkesmann [35]. Wilkesmann distinguishes between institutions (regulatory, cognitive, and normative rules) and structures (authoritative and allocative resources), which influence the actions of actors (micro level) at the meso level. This integration of theories was extended to the macro level. Cognitive rules refer to shared interpretive frameworks that structure how actors perceive reality and define what is taken for granted in everyday organizational life. In the analysis, cognitive rules capture how respondents make sense of work, workload, productivity, and the 4DW as plausible, desirable, or problematic. Examples of interview questions in this category include: “In your opinion, how does the general work culture in Germany view the idea of a four-day workweek in the healthcare sector?” (macro level) or “To what extent might social expectations within the hospital influence acceptance of the four-day workweek?” (meso level).

Normative rules capture value-based expectations and moral obligations that define what actors believe ought to be done. They are particularly salient in professional contexts such as healthcare, where ethical commitments and care norms play a central role. In this study, normative rules inform how actors justify their positions regarding the 4DW with reference to responsibility for patients, fairness between occupational groups, professional ethos, or the legitimacy of prioritizing self-care and employee well-being. Sample questions regarding normative rules include: “Would hospital staff consider a four-day workweek for nursing staff to be fair?” (meso level) or “How might your work on reduced four-day-week affect patient care?” (micro level).

Regulative rules denote formalized constraints and enablers of action, including laws, collective agreements, and organizational regulations. Analytically, they specify the formal boundaries within which different models of the 4DW can be conceived and implemented. In the empirical data, regulative rules are reflected in references to labor law, working-time regulations, staffing requirements, and collective bargaining agreements. Example questions are “What legal hurdles do you see in implementing a reduced four-day workweek in healthcare facilities?” (macro level) or “To what extent did company policies need to be adjusted to make the reduced four-day workweek work at the hospital?” (meso level).

Allocative resources refer to material and temporal resources such as staffing, time availability, financial resources, physical and mental energy, and technical infrastructure. Interview questions in this category are, for example, “How do you assess the economic situation of the healthcare sector in Germany with regard to the feasibility of introducing a reduced four-day workweek?” (macro level) or “You work part-time: Would you keep your current hours to earn a higher salary, or would you reduce your hours and continue to earn your current salary? What are your reasons?” (micro level).

Authoritative resources, on the other hand, derive from hierarchical positions and leadership structures that enable actors to coordinate, lead, or influence others. In the study, authoritative resources are defined in terms of decision-making powers, leadership authority, professional hierarchies, and the legitimacy to define priorities and acceptable forms of change. Interview questions for authoritative resources are, for example, “To what extent do current health policy reforms support the introduction of a reduced four-day workweek in Germany?” (macro level) or “How would existing company agreements need to be amended to accommodate a reduced four-day workweek? Would such changes be realistic?”.

Taken together, this analytical distinction allows the study to examine how interpretations of the 4DW are shaped not only by individual preferences but by the interplay of institutionalized rules and unevenly distributed resources across organizational levels. The interview guide initially focused solely on the R4DW. However, it became apparent what role the C4DW also plays for actors, which is why questions were added here.

The interview guide and the resulting interviews could be specifically utilized for further theoretical applications. In an initial analysis, the 4DW discussed in the interviews was evaluated in terms of environmental, economic, and social sustainability. These were deductive categories based on Carayannis and Campbell [36] and Blümel et al. [34]. “Organizational performance sustainability” was added as an inductive category. Thus, the focus of the first paper is on environmental, economic, and social sustainability, as well as on the tensions arising from the sustainability assessments [30].

This paper focuses on the various models of the 4DW as well as part-time work. The categories were formed deductively based on Cajaiba-Santana’s model of the development and institutionalization process [31]. Based on the theoretical framework, the three central dimensions (initiation, idea, and institutions) were first developed as top-level categories. These categories were then further subdivided into cognitive, normative, and regulative rules, as well as allocative and authoritative resources. The transcribed interviews were analyzed using MAXQDA software according to the qualitative structured content analysis of Mayring [37]. In this qualitative analysis method, the research question determines the direction of the analysis, that is, which content-related aspects are to be identified in the interviews. Building on this, a category system is developed based on theoretical considerations and the interview guide. As described earlier, the categories are cognitive, normative, and regulatory rules, as well as authoritative and allocative resources, each of which was examined at the micro, meso, and macro levels. For each category, definitions, anchor examples, and clear coding rules are established. The material is then systematically reviewed, and relevant text passages are assigned to the categories. Finally, the statements within the categories are described and interpreted.

The present analysis does not take into account the dimensions “social system” and “institutionalization,” as the available data are not suitable for this purpose. The “social systems” category aims, within the context of one or more case studies, to examine in detail how social practices, norms, and structures function and change within individual healthcare facilities. This would require conducting interviews with several individuals working at a specific facility in order to reconstruct the internal logic and dynamics of that organization in depth. The dataset presented here does not include any series of interviews focused on a specific healthcare facility. Instead, experts from various organizations were interviewed. An in-depth case study of individual facilities therefore represents a logical next step in the analysis of the 4DW, one that goes beyond the scope of the present study.

Rather than approaching the micro, meso, and macro levels as discrete domains, the present study examined how a single innovation claim can assume different meanings for different actors. Consequently, an exploratory investigation was conducted at the micro level, followed by a comprehensive analysis of its correlation to the meso and macro levels. Conversely, actions or problems at the micro level can be the result of decisions and structures at the meso and macro levels. Concurrently, actions and problems at the micro level can result in new courses of action and structures at the meso and macro levels.

**Fig. 1:**
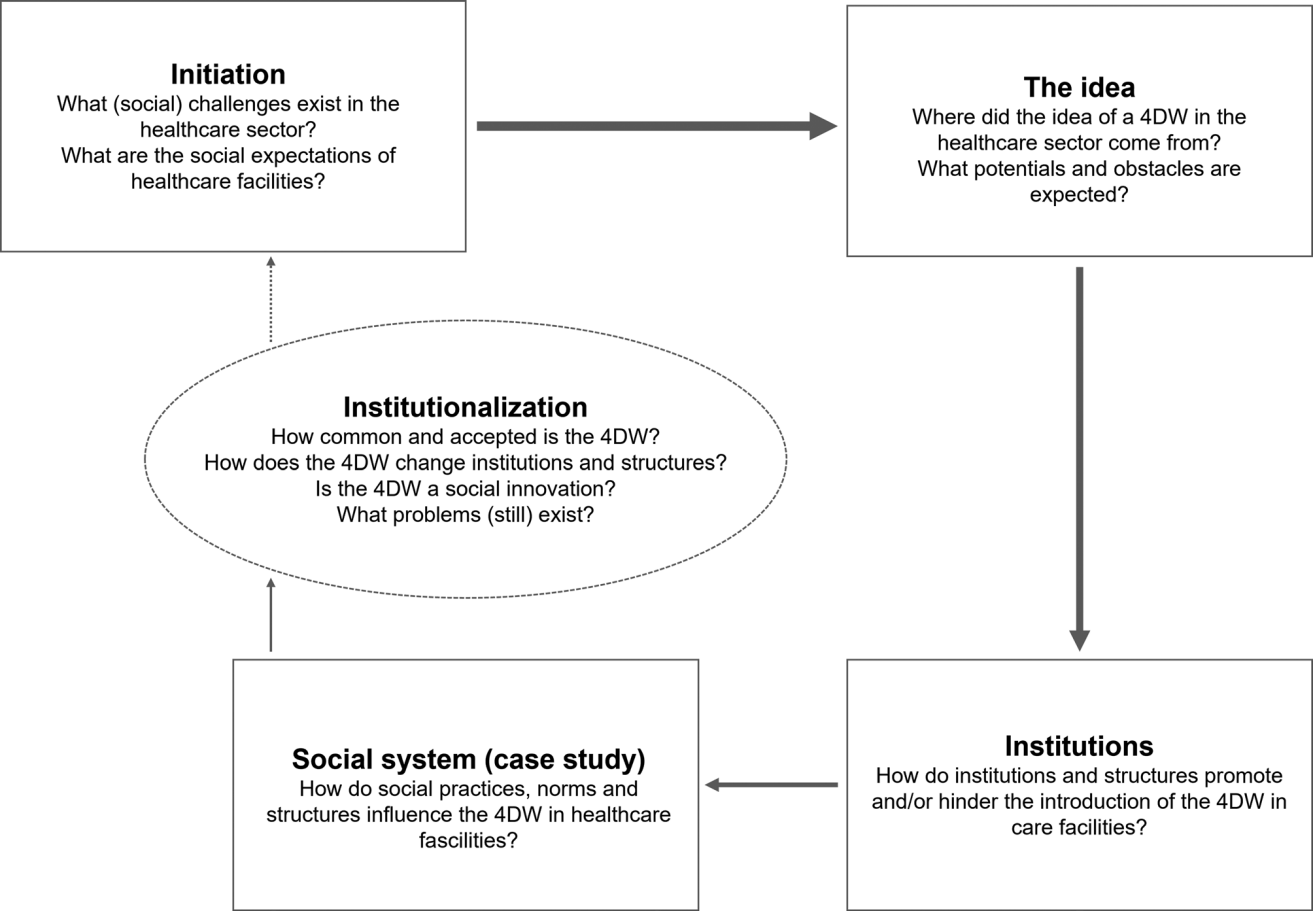
Development and institutionalization process of the 4DW as a social innovation based on Cajaiba-Santana [31]

The category system was developed in theory-driven: It was developed in accordance with the model proposed by Cajaibana-Santana et al. [31]. This model was then implemented in the 4DW (see Fig. 1) to identify the individual categories and the key questions associated with each category. In this step, categories include the initiation, the idea, and institutions. Social systems as a case study represent an alternative research design, in which the respective care facility must be examined in detail. At this juncture, it is not yet feasible to analyze the extent to which 4DW models have been institutionalized, as the models are still in the process of being implemented.

## Empirical findings

The results are presented according to the following categories: initiation, the idea, institutions, and the social system.

### Initiation of the 4DW models

To understand the 4DW models in hospitals, it is first necessary to reconstruct the central challenges in the healthcare system. These challenges arise from the tension between limited temporal, personnel, and financial resources and high expectations of good medical and nursing care. They shape rules, as well as the distribution of resources and thereby form the context in which new working-time models such as the 4DW emerge.

At the micro level, nurses understand their work as demanding but meaningful and tied to patient recovery:

I find it enriching when you have a lot of work with some patients who also need to be positioned, put to bed, and cared for. And over time, you see them getting out of bed on their own again, walking down the hallway on their own again, rehabilitating themselves, and you can calmly send them to rehab knowing that you have really done everything you can to help them get their old life back.

Teamwork is seen as a key resource and a precondition for coping with workload:

I really enjoy the teamwork. I’m not really a lone wolf. Thankfully, there are now two of us working at night. It’s just great. Of course, it has to be that way. Otherwise, you can’t get the work done. But we work hand in hand. It’s fun, I have to say. (Nurse 05)

The 24/7 character of hospital work is normalized as something that structures the whole life:

Well, I sometimes had to work three weekends a month. And then you couldn’t do anything in the evenings. And with the late shift, which ends at 10 p.m., it’s also a bit late. (Nurse 03)

At the same time, financial resources and their use are evaluated by the actors: There is a widespread perception that existing funds are used inefficiently and that investments concentrate on lucrative areas:

Of course, there is a large funding gap. A lot needs to be done in terms of construction, renovation, and also in university hospitals. Where a lot is being done is in the areas where a lot of money is being made. (Trade Union Secretary with a Focus on Youth Issues)

A big problem is that talking medicine and care do not have the status they should have. We spend far too much money on drugs and far too little on personal attention from doctors or nurses. […] In many areas, the focus is no longer on ensuring the best possible care, but on generating returns. (State Parliament Member with a Focus on Health and Social Policy 03)

Normative rules define what actors feel they ought to do. Nurses hold strong ideas of professional responsibility and “good care”, but these collide with staffing and skill mix:

And I work with ventilators and ventilated patients, and I don’t actually have any specific training in this area. It’s all learning by doing […]. Yes, and that’s also something that happens sometimes, that I take on tasks where I’m not 100% sure that I can do them. (Nurse 04)

Because of staff shortages, basic nursing is postponed in favor of life-sustaining measures, waiting times increase and continuity of care suffers. These developments contradict professional norms and create moral stress. At the same time, there are strong societal expectations of best possible care everywhere:

Ordinary citizens simply want to receive the best possible medical care. And that is exactly what we are trying to ensure – regardless of whether someone lives in a large city such as Cologne, Bonn, or Düsseldorf, or in a rural region such as the Hochsauerlandkreis or the Eifel. The standard is the same everywhere: the best medical care that is currently feasible. (State Parliament Member with a Focus on Health and Social Policy 03)

Normative commitments also underpin informal flexibility when staff fill in at short notice, although this is not formally required:

Colleagues still have to step in at short notice and swap shifts at short notice. We have also created tools for this purpose, to give value to these short-term swaps, i.e., this flexibility, which strictly speaking is not specified anywhere. […]. The idea is that if you fill in at short notice, you get a certain amount of money, so in addition to the time you get as a replacement anyway, you get an extra bonus for giving us your time, which you can then take off at some point. (Staff Council Representative and Physiotherapist)

The healthcare system is subject to a high degree of regulation, with the aim of ensuring the quality and quantity of patient care. A central element is the legal and contractual obligation to provide round-the-clock care, which establishes shift work as the standard. Working time guidelines, collective bargaining agreements, and personnel regulations set minimum staffing levels, maximum working hours, and rest periods.

At the same time, financing systems and billing rules create an additional layer of bureaucratic complexity and economic pressure. DRG-based financing links revenue to documented services and the case mix, while investment and operating costs are regulated by various funding agencies and programs.

The process of resource allocation is the transformation of these rules into specific guidelines regarding staff, time, money, and energy. Nurses report significant physical and psychological strain:

I find it a bit difficult to set these priorities sometimes. And then, when there is time pressure because of staff shortages, I have to decide what is more important, but it doesn’t affect me, it affects other people. […] And then I just have to live with the consequences. (Nurse 04)

But it is a psychological strain, especially when you can’t care for people the way you want to. When there are setbacks, when you make mistakes. Then, of course, you have to deal with it. There is conflict within the team. That’s what happens when there are fewer and fewer people and more and more work fall on your shoulders; the pressure keeps growing. And then you take things home with you. (Nurse 05)

At the meso level, staff shortages, underfunding, and the shift system exacerbate this depletion. The constant need to cover shifts has been shown to generate pressure to take on extra duties, thereby undermining reliable rest. For hospital leaders, ensuring an adequate staff is now the primary concern.

My job is primarily to recruit enough nursing staff here and, of course, to retain them. At a level that meets certain standards. In my opinion, that’s the biggest problem. The other issues are financial problems, the economic situation of hospitals, investment planning, and all those topics. But that’s all useless if you don’t have skilled workers. (Director of Nursing 03)

Directors of nursing describe their role as “managing shortages” (Director of Nursing 04) – allocative scarcity shapes everyday management.

Power dynamics are defined by the allocation of authority, the delineation of roles, and the initiation of change. Interprofessional relationships are of particular significance in this context:

Yes, sometimes doctors try to foist their tasks on us a little. And then you often have to make a clear distinction and say, sorry, but that’s clearly a doctor’s job. And they sometimes forget that. Or yes, they just try to make it happen. (Nurse 03)

When tasks are shifted downwards, role conflicts arise. These conflicts are indicative of cognitive rules concerning professional identities. These dynamics are influenced by the power dynamics inherent to specific groups. The shortage of skilled labor affects physicians as well, leading to informal task allocation between different professions. In turn, nurses attempt to allocate tasks to nursing assistants.

In addition to the challenges posed by substantial workloads and shifting responsibilities, other organizational shortcomings have been identified. These include a lack of supervision.: *We don’t get any supervision from the hospital. We don’t get any groups where we can talk. We have to leave on time. (Nurse 05).*

### The idea: How structures and institutions shape four-day week models in healthcare

In the following section, I will present a reconstruction of the manner in which various models of a 4DW are conceptualized and justified within the healthcare sector.

At the level of the individual employee, the response to high qualitative and quantitative workloads is often a reduction in working hours or even the complete abandonment of the profession. This applies to both nursing and the medical service:

So, what I have clearly observed over the last few years, and actually over the last few decades, is that the proportion of people working part-time is increasing. And that is a reflection of the fact that it’s not that people want to earn less money. (Chief Physician)

And now, if we turn to the medical sector, the collective agreements that we have fought for over the last few decades, which began in 2005 and 2006 with our Marburger Bund collective agreement, are now so well-funded for people that they can get by wonderfully even with a part-time job. […] And what you hear over and over again is simply that people say, I want to be done with 50 hours, so I’m only working three-quarters time, which is actually crazy when you think about it. (Chief Physician)

In nursing, part-time work is constructed as a necessary protection strategy, even though it entails a high risk of poverty in old age:

So, if I stick with 20 hours, stick with 20 hours, now until I reach retirement age, I won’t get anything. I’ll get 1,100€. What am I supposed to do with that? I can’t even go shopping with that. So, you’ll have to supplement it, and then you’ll notice. (Nurse 05)

Interviewees are aware of these risks, but they prioritize protecting their health over a higher salary. This makes part-time work and the 4DW appealing responses to an excessive workload.

Ideals of “good work” and “fair treatment” influence norms regarding working time. A R4DW, which has been tested mostly outside healthcare, is described as an expression of appreciation and a way to improve health, well-being, and work-life balance for employees. The advantages of an R4DW can therefore be classified as normative: work should enable a healthy life course and not lead to early exit from the profession.

A system like this could result in reduced absenteeism and longer career retention, which could lead to more efficient resource allocation. The additional free time resulting from reduced working hours could be allocated to social engagement. At the macro and meso levels, the profession’s appeal could increase, and staff retention could improve.

In the case of the C4DW, however, normative expectations diverge slightly. In this model, the total number of work hours remains constant, but they are distributed across four days. Interview participants identified employees’ desire for more consecutive days off and the appeal of a C4DW for certain life stages or lifestyles as important factors. Therefore, a C4DW is associated with the expectation that a modern employer should offer flexible work arrangements, even if the daily workload increases.

Regulations and laws are central to understanding the C4DW. Hospitals operate within a strict framework of continuous care, statutory staffing requirements, and Diagnosis-Related Group (DRG)-based funding. Within this framework, reducing the overall volume of hours and services is not as simple as it seems. Consequently, the C4DW model has been developed to adapt to existing regulatory rules rather than challenge them.

At the meso level, hospitals develop strategies for retaining staff while complying with legal and contractual obligations. One option is to reorganize the same amount of work time into fewer, longer shifts. This allows hospitals to comply with staffing, opening hours, and financing rules while responding to their employees’ wishes.

Nursing directors who introduced a C4DW considered existing work-time models outdated. They felt pressured to innovate and saw the C4DW as a competitive advantage over other hospitals:

We simply decided to do it because we feel it is appropriate at the moment to have this offer available to everyone. Keyword: en vogue and things like that. That was actually the main reason. Just to have the offer. Of course, this can also be supplemented, also with a view to the competitive situation here in the region, because no one else has implemented it yet. We wanted to be the first. (Director of Nursing 03)

Because the C4DW does not reduce total hours, it can be integrated into existing rotas and collective agreements with comparatively few changes. It fits into the regulative logic of full-time and part-time contracts and uses longer daily shifts as a legal way of „stretching“ staff coverage.

Regulations around documentation and billing also play a role. Longer overlap times in 10-hour shifts can be used to improve documentation and thus exploit funding rules more effectively:

What I achieve in terms of increased productivity is better documentation. That means I can bill more accurately, I know more precisely and perhaps more quickly how people are doing, and I can provide better patient care. This means that my outcome is ultimately better for the traditional customer who says, this is certainly an increase in productivity that society will also notice. If all hospitals made the change, it would of course be great for the healthcare system. (Director of Nursing 02)

In short, the C4DW is a way of rearranging working hours to ensure compliance with the 24/7 funding regime while signaling modernity and attractiveness.

At the macro level, reform measures aim to streamline the system and increase efficiency. Basic funding is intended to reduce misguided incentives, and specialization and reduced locations aim to lower staffing requirements. However, there is concern that nurses will leave the profession rather than move to other facilities in the event of closures.

Financial and personnel resources are particularly important for discussing an R4DW. From the interviewees’ perspective, the potential benefits of an R4DW include reduced absenteeism and longer career retention, i.e., stabilized personnel resources. Additional days off could help nurses regenerate physical and mental energy.

However, the greatest challenges identified by the interviewees are precisely allocative: staffing levels and funding:

But that would also require me to have more people to ensure care. Because that doesn’t change the fact that the issue is 24/7. […] And given the expected workload, we can’t avoid needing a certain number of staff. (Staff Council Representative and Physiotherapist)

In contrast, the C4DW does not increase the total number of hours, so it can be justified as “cost-neutral” by allocating the same hours differently. The potential allocative gains, such as better documentation, fewer handovers, and easier recruitment, are weighed against the risk of an intensified daily workload. The adaptation of the C4DW to regulations (same contracted hours and 24/7 coverage) makes it appear feasible under allocative constraints.

Finally, power dynamics determine who can initiate and shape 4DW models. At the meso level, nursing directors and management teams have the formal authority to implement the C4DW. They use their decision-making powers to reorganize working hours within existing legal and financial boundaries when doing so.

At the same time, bottom-up impulses are important. Management considers staff surveys and labor market developments when designing the models:

And in the surveys, apart from the fact that more money is always nice, of course, one of the findings was that the stress situation due to the increased workload and working hours is perceived as a disruptive factor. (Chairperson of a Regional District Association of a Non-profit Provider)

Overall, C4DW appears to be a flexible model that adapts to existing regulations and resources. In contrast, R4DW would require a fundamental reconfiguration of staffing and financing.

**Fig. 2:**
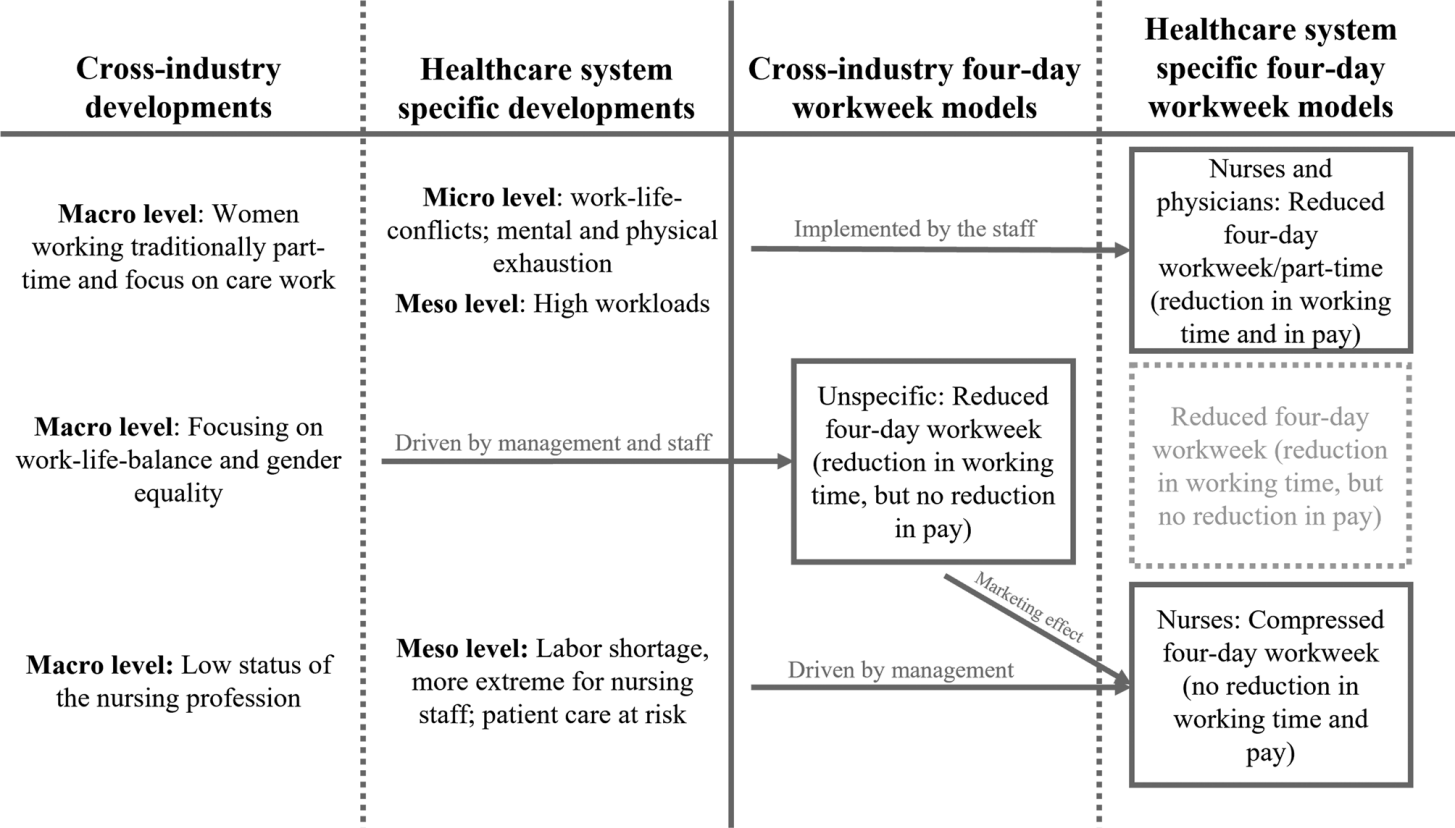
Developments of the 4DW-models cross-industry and healthcare system specific

Figure 2 shows the different types of 4DW (part-time, C4DW, and R4DW) in healthcare: Across society as a whole, women are more likely to work part-time and take on a larger share of caregiving responsibilities. At the same time, the importance of work-life balance has increased significantly in recent years. In the healthcare sector, the proportion of part-time employment is particularly high, especially among nursing staff. A key reason for this is the high workload, which is why the part-time model in this sector is predominantly employee-driven. In addition to these pressures, the nursing profession has long been associated with a comparatively low social status, which has further exacerbated the shortage of skilled workers. Against this backdrop, decision-makers in management increasingly recognized the need to take action. As a result, models such as the R4DW and the C4DW were introduced or intensively discussed for nursing staff. The figure illustrates which stakeholders drive the respective models (management- or employee-driven) and how developments in the healthcare sector are linked to broader societal trends.

### Institutions

Various institutional rules, norms, and values shape the existing system and influence how the 4DW models are perceived. For nurses, patient care is central to their professional identity. They are intrinsically motivated and responsible. However, this motivation has its limits under conditions of staff shortages. Many nurses are no longer able to meet their own standards, emphasizing that they could work with more concentration and empathy if they were better rested and less stressed:

And I don’t know if the work itself would change. Maybe your motivation would be even higher. Sometimes you’re just exhausted from work, depending on what kind of shifts you’ve had, how long you’ve been working in a row. I think that could be perceived as pleasant and therefore boost morale. (Nurse 04)

In addition, work–life balance is highly valued and becomes part of the cognitive framing of „good work“. A R4DW is therefore often seen as an opportunity to better align leisure, personal commitments and work:

Hobbies, exactly. Especially because most of my friends also work in hospitals, it’s very difficult to see each other. Because maybe you work staggered shifts all the time. Then you can’t meet up anywhere. But if you have a few more days off, you’re more likely to have time off at the same time or maybe have a weekend off. (Nurse 03)

Ideas about good care and a work–life-balance thus shape a preference for the reduced model. By contrast, physicians articulate a different professional self-image. For them, even a C4DW is cognitively framed as noticeable relief within existing long-hour cultures:

So now in the medical field, let’s say, if I apply that to myself personally, it’s a great idea. I’m at the clinic until seven anyway, so it doesn’t matter to me. But for the others who work, say, 40 h–38.5 h a week, it would be a completely different quality of life to say, “I finish at five on Thursday and then have three days off.” (Chief Physician).

In the context, value-based expectations and ideas of what ought to be done are very important. For nurses, these expectations concern both “good patient care” and fair distribution of recovery time. The desire to be well-rested in order to meet their own care standards links normative expectations to the R4DW. Fewer workdays are not only about personal comfort, but also about being able to provide proper care.

At the macro level, a central institution is responsible for ensuring nursing and medical care as public goods: *Ordinary citizens simply want to receive the best possible medical care. And that is exactly what we are trying to ensure […]. (State Parliament Member with a Focus on Health and Social Policy 02)*.

This normative claim to comprehensive and high-quality care places limits on models that could be seen as reducing performance, but it also strengthens the argument for working-time models that preserve staff health and thus long-term capacity.

Normative positions diverge when it comes to the R4DW. Trade union representatives and some politicians tend to favor it, as they interpret it as an instrument of relief and retention:

Yes, I can imagine that under certain parameters, [an R4DW] could also have economic advantages. […] But if I say that someone really only works four eight-hour shifts, then I would argue that people are more balanced, don’t get sick as often, and so on. And that, of course, also has economic advantages. We currently have a sickness rate of 20 to 25% in the healthcare sector. […] If we could reduce that, we would have a big advantage. Definitely. (Trade Union Secretary 02)

Some politicians, on the other hand, reject the R4DW and invoke a performance-oriented norm:

Yes, well, as I said, you get the impression that people don’t want to work much anymore and that the principle of performance is no longer seen as the top priority. Instead, I work and immediately start looking forward to the end of the day or my vacation. […] We can’t just live for our free time, and often you get the impression that people are so stressed out by all their free time that they can’t go to work anymore. (State Parliament Member with a Focus on Health and Social Policy 05)

Thus, normative rules at different levels either support the idea of a reduced model (as protection and appreciation) or problematize it as a departure from performance norms.

Regulations address the formal structures of financing, responsibilities, and coverage requirements. At the meso level, managers’ decisions are influenced by institutions considered legitimate and binding within the organization. Most managers prefer the C4DW because it can be more easily integrated into existing organizational and financial structures. The C4DW is compatible with staffing requirements, collective agreements, and funding logic because it reorganizes working time without formally reducing it. This adaptation to the existing regulatory framework makes the compressed model more acceptable.

It’s just nicer to advertise a four-day week than 9-hour shifts. That’s why we have the four-day week. […] And the effect on documentation, patient care, and training is extremely positive. So, things are really happening there. (Director of Nursing 01)

For nursing directors, the C4DW is less controversial on a normative level because it does not require reducing total working hours, which would jeopardize continuity of care and economic viability.

At the macro level, the healthcare financing system structures the distribution of resources and responsibilities. Reform efforts aim to make the system more efficient by changing funding incentives, concentrating services, and reducing the number of locations. While these reforms aim to reduce costs and staffing requirements, they also raise fears that nurses will leave the profession rather than move to other facilities.

Thus, regulatory rules both limit and enable: they restrict the scope of a R4DW while making the compressed model attractive as a “rule-conforming” innovation.

**Allocative resources** are closely intertwined with these rules. The tension between normative expectations of good care and limited personnel resources becomes particularly visible where nurses describe their exhaustion and the hoped-for gains from more recovery time:

And I don’t know if the work itself would change. Maybe your motivation would be even higher. Sometimes you’re just exhausted from work, depending on what kind of shifts you’ve had, how long you’ve been working in a row. I think that could be perceived as pleasant and therefore boost morale. (Nurse 04)

An R4DW is associated with potential benefits, such as reduced absenteeism and longer career retention. However, interviewees also point out significant financial risks and uncertainties. It is difficult to estimate staffing needs and personnel costs under an R4DW. Many initially expect cost increases of around 20%. In a predominantly publicly funded system, questions arise about whether financing and comprehensive coverage could be maintained and whether the system would be socially accepted.

These concerns are underscored by structural limits in training and recruitment:

The nursing profession is the profession that most students end up in. […] And we won’t be able to expand that. Nor will we be able to compensate for the demand by recruiting from abroad, which is already being done on a large scale. That’s why I think it will be very difficult to implement this from a macroeconomic perspective. […] Then we’ll just have to see what nursing is ultimately worth to us and whether we can still manage to care for people. If we can do that, then we can talk about it. But that’s the trade-off that has to be made. (State Parliament Member with a Focus on Health and Social Policy 02)

In many cases, the C4DW results in a 10% increase in personnel costs due to longer overlap periods. However, under the current financing system, in which health insurance companies reimburse nursing staff, this increase is easily manageable because the hourly wage for nursing staff does not increase.

Power dynamics refer to the ability of actors and institutions to enforce decisions, define priorities, and legitimize models. At the organizational level, nursing directors and hospital managers have the authority to implement working-time models, such as the C4DW, and present them as modern and successful. Their preference for solutions that fit into existing regulatory and allocative constraints lends particular weight to the compressed model because it is presented as both innovative and “responsible.” Power dynamics also operate in everyday professional hierarchies. Medical and managerial positions can support or impede experiments by approving pilots, allocating project roles, or insisting on traditional rota systems, for example. At the macro level, political actors and collective organizations wield power by defining what is publicly acceptable and financially justifiable. Concerns about the public acceptance and macroeconomic feasibility of an R4DW demonstrate the extent to which the debate is linked to these authoritative instances.

Overall, from an institutional perspective, the C4DW currently appears as a model that can be integrated with manageable effort, as it largely adapts to existing cognitive, normative, and regulative structures. By contrast, the R4DW pushes more strongly against these structures and would require broader shifts in financing, staffing, and public expectations in order to become part of the healthcare “social system” in the long term. Whether and how either model will ultimately become institutionalized remains uncertain, as the future development of new working time models in the healthcare sector is still unclear.

**Fig. 3:**
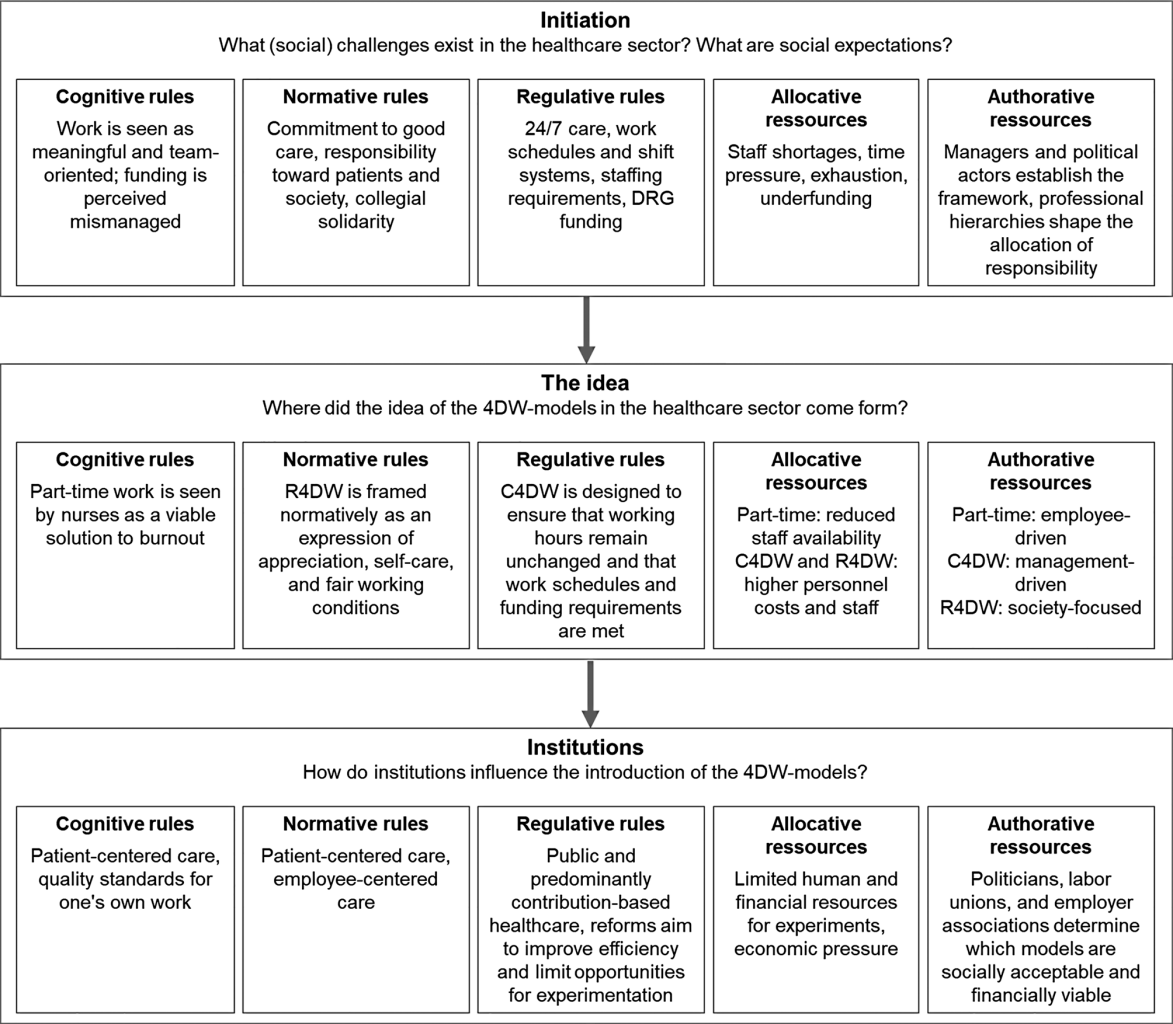
Own presentation of the empirical findings based on the model of institutionalization of social innovations by Cajaiba-Santana [31]

This highlights a broader institutional mechanism: while the C4DW exemplifies a logic of institutional adaptation or translation, where innovations are reshaped to fit existing rules, the R4DW represents potential path-breaking or reconfigurative change. Thus, the healthcare field illustrates how path dependencies and regulative constraints steer organizational innovation towards incremental rather than transformative forms of change. Whether reduced models like the R4DW can gain legitimacy will depend on their ability to align with the prevailing institutional logics of care, efficiency and fiscal responsibility. The following Fig. 3 illustrates the results described above.

## Discussion

### The 4DW-models as potential social innovations

One of the central questions this paper addresses is the extent to which the various models have the potential to serve as social innovations. Part-time work, which involves reduced working hours and lower pay, is an important model for understanding the C4DW and R4DW models. However, part-time work does not represent a new working time model across industries or in the healthcare sector specifically. Employees primarily use part-time work to cope with high workloads. Actors at the meso and macro levels allow this practice, but they do not actively promote it. In contrast, the C4DW and R4DW models are measures implemented by meso-level actors. Both aim to increase the attractiveness of professions, improve work-life balance, and reduce workloads. While the C4DW has primarily been introduced for nursing staff, the R4DW generally targets all employees in organizations that follow this model. Empirical results show differences in their social innovation potential. The C4DW does not constitute social innovation in the strict sense. Although it is attractive to individual nurses because it can be better integrated into their private lives, it does not lead to any real relief. Work intensity remains high, and the scope of work remains unchanged. Consequently, hospitals continue to report staffing challenges despite the C4DW. Physicians assess the potential for relief more positively; however, the overall effect remains limited. Nurses rate the potential of an R4DW more highly but point out that it would require comprehensive restructuring at the meso level. Since part-time work is already used as an individual response to high workloads in the healthcare sector, one could assume that the R4DW would also provide relief.

### Translation instead of transformation: why the 4DW remains partial

The results show that the C4DW can be integrated into existing structures relatively easily, while the R4DW has transformative potential but is much more challenging from a systemic perspective. Actors at the meso level have only limited scope for action within the existing system to offer working time models that actually lead to a sustainable reduction in the workload of clinical staff. So far, actors at the meso level have been unable to absorb the tensions between patient care, economic pressure; instead, they pass them on to actors at the micro level.

The study also contributes to research on healthcare organization by explaining why strategies to enhance attractiveness, such as the 4DW, do not automatically alleviate systemic pressures but can even exacerbate existing tensions under certain conditions. Current challenges in healthcare arise from a conflict between normative rules and allocative resources. Values and norms exist among individual employees, within the organization, and at the societal level, and at each level they conflict with scarce financial and human resources. Cognitive and regulatory rules, as well as authoritative resources, constantly attempt to balance this conflict.

The findings confirm that healthcare institutions are characterized by hybrid and sometimes contradictory institutional logics [38], including care, efficiency, stability, self-care, and professionalism. The 4DW exacerbates these tensions by challenging existing value systems and pitting them against one another. Empirical evidence shows that these competing logics must be continuously negotiated in everyday organizational life and that change processes can only be successful if they address multiple levels simultaneously. Furthermore, the study illustrates that collaboration in the healthcare sector is strongly influenced by structural and institutional contexts [39]. Constellations of actors are embedded in sector-specific norms, governance structures, and resource logics that influence how problems are interpreted and which solutions are considered legitimate or feasible. Coordination problems thus arise not only from different professional cultures. They also arise from institutional environments. This interplay structures expectations, routines, and power relations. Against this backdrop, the R4DW can be classified as a social innovation in the sense of Cajaiba-Santana [31]. It is currently in a phase of emergence and partial local dissemination but has not yet taken the step toward broad institutionalization. Comprehensive implementation has not yet taken place, as it is not structurally anchored in the financing system or in collective bargaining or political frameworks.

### Implications

In practice, the results suggest that the introduction of newer workplace innovations in healthcare facilities should occur gradually. One possible approach would be to test models locally, iteratively, and reversibly in order to gain experience while simultaneously ensuring the continuity of care. At the same time, new work schedules must not further intensify the workload during the hours that remain. A study by Güngör & Sönmez [5] already suggests that there is a link between work intensification and fatigue. Furthermore, it is clear that workplace innovations are hardly feasible without clear political and financial frameworks. Appropriate refinancing mechanisms are necessary, for example through legal regulations, surcharges, or structural funding instruments. Pilot projects, iterative implementation, and participatory approaches involving healthcare professionals may help to test and refine new models while maintaining quality of care. Existing studies show that nursing staff want more opportunities to make decisions and feel disconnected from political decisions [7].

Collective bargaining agreements would also need to be further developed, as unions play a central role in negotiating issues of fairness and sector-specific solutions. However, healthcare workers need more than just new work-time models. Comprehensive internal support processes are required. These include the optimization of work and care processes, particularly through the digitization of documentation, as well as services such as supervision and psychosocial support. Such decisions must be negotiated and communicated transparently to foster acceptance and avoid inequalities.

### Limitations

The study has several limitations. Despite a sample of 27 interviews, the focus is on stakeholders from hospitals and politics, which limits the generalizability of the results. In addition, the empirical implementation of the 4DW in the healthcare sector has been low overall to date. Many findings are therefore based on individual experiences or assessments and do not allow for reliable statements about long-term effects on staff retention, quality of care, patient outcomes, or economic developments.

Furthermore, the debate on reducing working hours is highly charged politically, normatively, and emotionally, which may influence the perspectives of the interviewees. Finally, comparability between hospitals and other care facilities is limited, as organizational logics, financing models, and working conditions differ significantly. The results are therefore not readily transferable to other sectors.

## Conclusion

This study examined different models of the 4DW in the German healthcare system through the lens of social innovation. The findings demonstrate that working-time innovations do not unfold in a linear or purely transformative manner but are shaped by existing regulatory frameworks, resource constraints, and competing institutional logics.

The C4DW emerges as a pragmatic and adaptive solution that aligns with current organizational and financial structures. Its relative ease of implementation explains its growing diffusion in practice. However, its impact remains limited, as it primarily redistributes working hours rather than reducing workload, and may even intensify daily strain. In contrast, the R4DW reflects a stronger normative orientation toward employee well-being, health, and sustainable careers. While it holds greater potential to address structural challenges such as burnout and staff shortages, it faces significant barriers related to staffing capacity, financing mechanisms, and the requirement to continuous patient care.

Across all models, the analysis reveals persistent tensions between the goal of high-quality patient care and the scarcity of human and financial resources. These tensions manifest differently at the micro-, meso-, and macro-levels but are closely interconnected. As a result, innovations such as the 4DW often lead to paradoxical outcomes: they may improve individual well-being in the short term while simultaneously reinforcing systemic pressures or shifting burdens within the system.

Based on the understanding of social innovations as context-dependent practices that are shaped by interpretation and implementation [32], the analysis of the 4DW as a social innovation demonstrates the extent to which innovations can be reinterpreted during the implementation process. What is conceptually designed as a potentially transformative solution is reinterpreted in organizational practice and thus loses some of its original meaning.

The future of the 4DW in healthcare will depend on its ability to reconcile the competing demands of efficiency, care quality, and employee well-being. Without broader structural reforms, its potential as a social innovation will remain only partially realized.

## Supplementary Information

Below is the link to the electronic supplementary material.

Supplementary Material 1

## Data Availability

The datasets used and/or analyzed during the current study are available from the corresponding author on reasonable request.

## Abbreviations

4DW: Four-day workweek
C4DW: Compressed four-day workweek
R4DW: Reduced four-day workweek
