# Supplementary material for: Exploring the different models of the four-day workweek as social innovations in Germany’s healthcare system
Source: BMC Health Serv Res. 2026 Jul 14;26:978. doi: 10.1186/s12913-026-15099-5 (PMC13371269; doi:10.1186/s12913-026-15099-5)
Supplement: Supplementary file 1 — Supplementary Material 1 [file 12913_2026_15099_MOESM1_ESM.docx]

**Interview Guides: Exploring the Four-Day Workweek in German Healthcare**

Katharina Hast

**Interview Guide 1: Stakeholder in the Healthcare Sector**

| **Questions** | **Rule and/or Resources** |
| --- | --- |
| **1 Introduction** | |
| - Could you briefly introduce yourself? - What is your professional background) - What is your current position? - Could you briefly tell us about your employer? | personal information |
| **2 Macro level** | |
| - In your opinion, what are the major challenges currently facing the healthcare system? - Which of these challenges do you face on a daily basis? | allocative resources |
| - In your opinion, what are society’s expectations of stakeholders in the healthcare system and healthcare facilities? | normative rules |
| - Where would you say the idea of a four-day workweek in the healthcare sector comes from? What developments preceded it? | allocative resources, normative rules |
| - What potential benefits or improvements could arise at the health policy and societal levels regarding the introduction of a four-day workweek in the healthcare sector? - What challenges might arise at the health policy and societal levels regarding the introduction of the four-day workweek in the healthcare sector? - What are the inhibiting factors at the societal level that might influence the introduction of the four-day workweek? - What are the enabling factors at the societal level that might influence the introduction of the four-day workweek? | general |
| - What legal hurdles do you see in implementing a four-day workweek in healthcare facilities? - Which labor law provisions might support the implementation of a four-day workweek in the healthcare sector? | regulative rules |
| - How do current health policy reforms support the introduction of the four-day workweek? - How do current health policy reforms hinder the introduction of the four-day workweek? | regulative rules, allocative resources |
| - How do you assess the economic situation in the healthcare sector with regard to the feasibility of introducing a four-day workweek? - What financial challenges do you foresee in implementing this? - What economic benefits could result from its introduction? | allocative resources |
| - In your opinion, how does the general work culture in Germany view the idea of a four-day workweek in the healthcare sector? | cognitive rules |
| - How do you perceive public opinion regarding the four-day workweek in the healthcare sector? (normative rules, authoritative sources) | cognitive and normative rules, authorative resources |
| - How would you assess the attitudes of various stakeholders toward the four-day workweek? (health insurance companies, labor unions, politicians, hospital representatives, etc.) | authorative resources |
| - To what extent would you consider the four-day workweek to be fair on societal level? | normative rules |
| - How might digitalization play a role in the implementation of the four-day workweek in the healthcare sector? | allocative resources |
| **2 Meso level**  (Whenever the four-day workweek was discussed, both models (reduced and compressed) were always requested) | |
| - What challenges do healthcare facilities currently face? - What improvements could healthcare facilities see with the introduction of the four-day workweek? - What challenges might arise as a result? - In your opinion, what factors in a hospital influence the success of the four-day workweek? | general |
| - To what extent might social expectations within the hospital influence acceptance of the four-day workweek? - How does the work environment affect the success of a four-day workweek? - How might the four-day workweek change the work environment? | cognitive rules |
| - How would the four-day workweek need to be implemented in healthcare facilities so that it is perceived as fair by hospital staff? - Would hospital staff consider a four-day workweek for nursing staff to be fair? - How might a four-day workweek affect patient care? | normative rules |
| - What staffing measures are necessary to ensure that the four-day workweek works in hospitals? - What must managers do to ensure that the four-day workweek succeeds in healthcare facilities? | authorative resources |
| - How would you assess the financial impact on utility companies? - To what extent would you assess the financial feasibility? | allocative resources |
| - In your opinion, what criteria should be used to evaluate the success of the four-day workweek? | General, cognitive rules |
| **4 Micro level**  (Whenever the four-day workweek was discussed, both models (reduced and compressed) were always requested) | |
| - What are the benefits of the four-day workweek for employees? - How do you think a four-day workweek affects employees? - What challenges might employees face as a result of the four-day workweek? | general |
| - What factors might influence employees’ acceptance of the four-day workweek? | cognitive rules |
| - What specific health benefits do you see for employees? - What specific health risks do you see for employees? | allocative resources, normative rules |
| - What is your personal opinion on the four-day workweek? | general |
| **5 Closing** | |
| - Is there anything else you'd like to say about the four-day workweek? | general |

**Interview Guide 2: Nursing staff**

| **Questions** | **Rule and/or Resources** |
| --- | --- |
| **1 Introduction** | |
| - Could you briefly introduce yourself? - What is your professional background) - What is your current position? - Could you briefly tell us about your employer? | personal information |
| - In your opinion, what are the major challenges currently facing the healthcare system? | allocative resources |
| - In your opinion, what are society’s expectations of stakeholders in the healthcare system and healthcare facilities? - What expectations do patients have of you, your position, and the hospital? | normative rules |
| **2 Micro level: workloads** | |
| - What do you enjoy most about your work? What do you find particularly rewarding about your job? - What motivates you in your current position? - What makes your work challenging? What kinds of stress do you experience at work? - What changes to your work hours or shift schedule would make your job less stressful? | allocative resources |
| **3 Meso level: workloads** | |
| - In your opinion, what specific factors contribute to psychological stress in the workplace? - What working conditions lead to the nursing profession being viewed as unattractive and result in high stress levels for nursing staff? - In which tasks or processes do you think you work inefficiently? - Where do you see opportunities to save time on tasks or processes? - Are there tasks where digital or technical solutions cause additional stress? If so, which ones? - How could digital or technical solutions help make your work easier? | allocative resources |
| **4 Micro level: Four-day workweek**  (Whenever the four-day workweek was discussed, both models (reduced and compressed) were always requested) | |
| - What is your general opinion on a four-day workweek? | general |
| - What would a four-day workweek mean for you, or how would it change things for you? - How do you think a four-day workweek would affect your work? - Do you think a four-day workweek could reduce your stress levels? Why or why not? - If there were a four-day workweek, would you want to adjust your work schedule? - You work part-time: Would you keep your current hours to earn a higher salary, or would you reduce your hours and continue to earn your current salary? What are your reasons? - Would a four-day workweek influence your professional future? - What benefits could a four-day workweek have for you personally? - How would a four-day workweek affect your personal life? - What would you do with the extra free time? - What hobbies or interests would you like to pursue more if you had extra free time? - What drawbacks could a four-day workweek have for you personally? | allocative resources |
| **5 Meso level:** **Four-day workweek**  (Whenever the four-day workweek was discussed, both models (reduced and compressed) were always requested) | |
| - To what extent do you think a four-day workweek would be feasible at your hospital? | cognitive rules |
| - What is important to consider when planning a four-day workweek? - What staffing measures would be necessary? - What responsibilities should managers take on when planning a four-day workweek? | allocative resources, authorative resources |
| - How do you think reduced working hours would affect your colleagues’ work? - How might a four-day workweek influence team spirit or collaboration? | cognitive rules, allocative resources |
| - How should the four-day workweek be structured so that all employees consider it fair? - What factors could influence acceptance of the four-day workweek? - How would the four-day workweek affect the workplace atmosphere? | normative rules, cognitive rules |
| - What improvements could a four-day workweek bring to healthcare facilities? - What challenges might arise as a result? - What criteria should be used to evaluate the success of the four-day workweek? - In what other shift models do you see benefits? | general |
| **6 Macro level:** **Four-day workweek**  (Whenever the four-day workweek was discussed, both models (reduced and compressed) were always requested) | |
| - To what extent do you think a four-day workweek is feasible across the board in healthcare facilities? - A four-day workweek with reduced working hours would be more expensive—what is your opinion on this? - To what extent do you think a four-day workweek could help address the shortage of skilled workers in the healthcare sector? | allocative resources |
| - How do you assess public acceptance of a four-day workweek? | authorative resources |
| **7 Closing** |  |
| - Is there anything else you'd like to say about the four-day workweek? | general |
